# Supplementary material for: Potential gains in life expectancy from reducing amenable mortality among people diagnosed with serious mental illness in the United Kingdom
Source: PLoS One. 2020 Mar 27;15(3):e0230674. doi: 10.1371/journal.pone.0230674 (PMC7100972; doi:10.1371/journal.pone.0230674)
Supplement: S1 File — (DOCX) [file pone.0230674.s001.docx]

**Supplementary material**

1. Table S1 Supplementary material describing the sources for study estimates regarding PAR calculation and effect size adjustment
2. Table S2 Potential gains in life expectancy at birth and ages 50 and 65 years associated with modifiable risk factors for all-cause mortality in specific SMIs.

**Table S1 Supplementary material describing the sources for study estimates regarding PAR calculation and effect size adjustment**

|  | Risk factor | Year | Design^a^ | Schizophrenia (%) | Bipolar (%) | Schizoaffective (%) |
| --- | --- | --- | --- | --- | --- | --- |
| **PREVALENCE** | | | | | | |
| ***Behavioural determinants*** | | | | | | |
| Janney et al.(1, 2) | Sedentary behaviour | 2014 | Observational | 81 | 78 | 81 |
| Mangurian et al.(3) | Smoking | 2016 | Meta-analysis | 59 |  | 59 |
| Jackson et al.(4) | Smoking | 2015 | Meta-analysis |  | 49 |  |
| Vancampfort et al.(5) | Obesity (central) | 2015 | Meta-analysis | 50 | 50 | 50 |
| Jakobsen et al.(6) | Diet | 2018 | Observational | 69 | 69 | 69 |
| Hunt et al.(7) | Substance abuse | 2016 | Meta-analysis |  | 45 |  |
| Viron et al.(8) | Substance abuse | 2010 | Meta-analysis | 47 |  |  |
| Green et al.(9) | Substance abuse | 2005 | Meta-analysis |  |  | 54 |
| Vancapmfort et al.(5) | Metabolic syndrome | 2015 | Meta-analysis | 33 | 32 | 35 |
| ***Social determinants*** | | | | | | |
| O’Donoghue et al.(10) | Deprivation | 2016 | Meta-analysis | 30 |  |  |
| Kwok et al.(11) | Deprivation | 2014 | Meta-analysis |  | 26 | 26 |
| Hawke et al.(12) | Stigma | 2013 | Meta-analysis |  | 55 |  |
| Gerlinger et al.(13) | Stigma | 2013 | Meta-analysis | 65 |  |  |
| Viron et al.(8) | Stigma | 2010 | Meta-analysis |  |  | 49 |
| Da Rocha et al.(14) | Social exclusion | 2018 | Meta-analysis | 32 |  | 32 |
| Gayer-Anderson et al. (15) | Social exclusion | 2013 | Meta-analysis |  | 21 |  |
| ***Healthcare determinants*** | | | | | | |
| O’Brien et al.(16) | Healthcare access | 2009 | Meta-analysis | 46 | 46 | 46 |
| Mitchell and Lawrence et al.(17) | Revascularisation | 2011 | Meta-analysis | 47 | 47 | 47 |
| Baller et al.(18) | Screening | 2015 | Meta-analysis | 50 | 50 | 50 |
| Patel et al.(19) | Clozapine | 2014 | National audit | 24 |  | 24 |
| Cipriani et al.(20) | Lithium | 2013 | Meta-analysis |  | 39 |  |
|  |  |  |  |  |  |  |
| **EFFECTIVENESS** | | | | | | |
| ***Behavioural determinants*** | | | | | | |
| Peckham et al.(21) | Smoking cessation | 2017 | Meta-analysis | 36 | 36 | 36 |
| Ashdown-Franks et al.(22) | Sedentary behaviour | 2018 | Meta-analysis | 25 | 25 | 25 |
| Teasdale et al.(23) | Diet | 2014 | Meta-analysis | 31 | 31 | 31 |
| Naslund et al.(24) | Weight loss | 2017 | Meta-analysis | 24 | 24 | 24 |
| Hunt et al.(25) | Substance abuse | 2013 | Meta-analysis | 17 | 17 | 17 |
| Tidey et al.(26) | SUD | 2012 | Meta-analysis | 35 | 35 | 35 |
| Tosh et al.(27) | Metabolic | 2014 | Meta-analysis | 30 | 30 | 30 |
| ***Healthcare determinants*** | | | | | | |
| Vermeulen et al.(28) | Clozapine | 2018 | Meta-analysis | 38 |  | 38 |
| Cipriani et al.(20) | Lithium | 2013 | Meta-analysis |  | 44 |  |
| Tosh et al.(27) | Speciality treatment | 2014 | Meta-analysis | 37 | 37 | 37 |
| Lamontagne-Godwin et al.(29) | Uptake of screening | 2018 | Meta-analysis | 41 | 41 | 41 |
| Liu et al.(30) | Healthcare access | 2017 | Meta-analysis | 38 | 38 | 38 |
| ***Social determinants*** | | | | | | |
| Corrigan et al(31)/Tsang et al.(32) | Stigma | 2012/2016 | Meta-analysis | 24 | 24 | 24 |
| McDonagh et al.(33) | Deprivation | 2017 | Meta-analysis | 23 | 23 | 23 |
| Anderson et al(34) | Soc exclusion | 2015 | Meta-analysis | 46 | 46 | 46 |
|  |  |  |  |  |  |  |
| **MORTALITY** | | | | | | |
| ***Behavioural determinants*** | | | | | | |
| Beary et al.(35) | Sedentary behaviour | 2012 | Meta-analysis | 1.66(1.59-1.74) | 1.66(1.59-1.74) | 1.66(1.59-1.74) |
| Callaghan et al(36) | Smoking | 2014 | Observational | 2.45(2.41-2.48) | 1.57(1.53-1.62) | 2.45(2.41-2.48) |
| Bellavia et al(37) | Low fruit/veg intake | 2013 | Observational ^b^ | 1.53(1.19-1.99) | 1.53(1.19-1.99) | 1.53(1.19-1.99) |
| Chwastiak et al(38) | Obesity | 2010 | Observational | 1.47(1.45-1.50) | 1.47(1.45-1.50) | 1.47(1.45-1.50) |
| Hjorthoj et al(39) | Substance abuse | 2014 | Observational | 1.78(1.56-2.04) | 1.89(1.34-2.66) | 1.78(1.56-2.04) |
| Correll et al(40) | Metabolic | 2017 | Meta-analysis | 1.95(1.41-2.70) | 1.57(1.28-1.93) | 1.95(1.41-2.70) |
| ***Healthcare determinants*** | | | | | | |
| Wimberley(41) | Clozapine | 2017 |  | 1.88(1.16-3.05) |  | 1.88(1.16-3.05) |
| Lewitzka et al(42) | Lithium | 2015 | Meta-analysis |  | 1.70(1.2-2.60) |  |
| Copeland et al.(43)/Mitchell et al.(44) | Healthcare quality | 2009 | Meta-analysis | 1.34(1.01-1.67) | 1.11(1.02-1.20) | 1.34(1.01-1.67) |
| Mitchell & Lawrence(17) | Treatment speciality | 2011 | Meta-analysis | 1.15(1.02-1.29) | 1.15(1.02-1.29) | 1.15(1.02-1.29) |
| Bergamo et al(45) | Screening | 2012 | Observational | 1.07(1.01-1.13) | 1.07(1.01-1.13) | 1.07(1.01-1.13) |
| ***Social determinants*** | | | | | | |
| Kilbourne et al(46) | Deprivation | 2017 | Observational | 1.36(1.29-1.45) | 1.36(1.29-1.45) | 1.36(1.29-1.45) |
| Kilbourne et al(46) | Social exclusion | 2009 | Observational | 1.19(1.14-1.25) | 1.19(1.14-1.25) | 1.19(1.14-1.25) |
| Barnes et al(47) | Stigma | 2008 | Observational | 1.12(1.04-1.20) | 1.12(1.04-1.20) | 1.12(1.04-1.20) |

Note: ^a^ Some of the meta-analyses were rather systematic reviews but for ease of interpretation were recorded here as meta-analyses.  ^b^ Based on general population data.

**Table S2** Potential gains in life expectancy at birth and ages 50 and 65 years associated with modifiable risk factors for all-cause mortality in specific SMIs. Confidence intervals for Life years gained (LYG) available on request.

|  | **Schizophrenia** | | | | |  | **Bipolar disorders** | | | | | | **Schizoaffective disorders** | | | |
| --- | --- | --- | --- | --- | --- | --- | --- | --- | --- | --- | --- | --- | --- | --- | --- | --- |
|  | **ES (%)** | **PAF (%)** | **LYG - age**  **Birth 50 65** | | |  | **PAF (%)** | **LYG- age**  **Birth 50 65** | | |  | **PAF (%)** | **LYG- age**  **Birth 50 65** | | |  |
| **LIFESTYLE MODEL** |  |  |  |  |  |  |  |  |  |  |  |  |  |  |  |  |
| **Smoking** | 36 | 46 | 2.3 | 1.9 | 1.5 |  | 22 | 1.0 | 0.8 | 0.7 |  | 46 | 2.3 | 1.9 | 1.5 |  |
| **Sedentary** | 25 | 35 | 1.2 | 0.9 | 0.7 |  | 34 | 1.2 | 0.9 | 0.7 |  | 35 | 1.2 | 0.9 | 0.7 |  |
| **Diet** | 31 | 27 | 1.0 | 0.8 | 0.7 |  | 27 | 1.0 | 0.8 | 0.7 |  | 27 | 1.0 | 0.8 | 0.7 |  |
| **Obesity** | 24 | 19 | 0.6 | 0.5 | 0.4 |  | 19 | 0.6 | 0.5 | 0.4 |  | 19 | 0.6 | 0.5 | 0.4 |  |
| **Substance abuse** | 17 | 27 | 0.6 | 0.5 | 0.4 |  | 29 | 0.6 | 0.5 | 0.4 |  | 30 | 0.6 | 0.5 | 0.4 |  |
| **COMBINED^b^** |  | **85** | **4.9** | **3.9** | **3.1** |  | **78** | **3.4** | **2.5** | **2.3** |  | **85** | **4.9** | **3.9** | **3.1** |  |
| **Metabolic syndrome** | **30** | 27 | 1.0 | 0.8 | 0.7 |  | 18 | 0.6 | 0.5 | 0.4 |  | 28 | 1.0 | 0.8 | 0.7 |  |
| **COMBINED (lifestyle)^a^** |  | **89** | **5.**3 | **4.2** | **3.4** |  | **82** | **3.3** | **2.5** | **2.2** |  | **89** | **5.3** | **4.3** | **3.5** |  |
|  |  |  |  |  |  |  |  |  |  |  |  |  |  |  |  |  |
| **HEALTHCARE MODEL** |  |  |  |  |  |  |  |  |  |  |  |  |  |  |  |  |
| **Clozapine/Lithium^b^** | 13/44 | 17 | 0.3 | 0.2 | 0.2 |  | 17 | 1.0 | 0.8 | 0.7 |  | 17 | 0.3 | 0.2 | 0.2 |  |
| **Healthcare access** | 38 | 14 | 0.7 | 0.6 | 0.5 |  | 5 | 0.3 | 0.2 | 0.2 |  | 14 | 0.7 | 0.6 | 0.5 |  |
| **Treatment disparity** | 37 | 7 | 0.4 | 0.3 | 0.2 |  | 7 | 0.4 | 0.3 | 0.2 |  | 7 | 0.4 | 0.3 | 0.2 |  |
| **Screening uptake** | 41 | 8 | 0.4 | 0.3 | 0.2 |  | 8 | 0.4 | 0.3 | 0.2 |  | 8 | 0.4 | 0.3 | 0.2 |  |
| **COMBINED** |  | **37** | **0.7** | **0.5** | **0.4** |  | **36** | **0.8** | **0.6** | **0.5** |  | **37** | **0.7** | **0.5** | **0.4** |  |
|  |  |  |  |  |  |  |  |  |  |  |  |  |  |  |  |  |
| **SOCIAL MODEL** |  |  |  |  |  |  |  |  |  |  |  |  |  |  |  |  |
| **Social deprivation** | 23 | 21 | 0.6 | 0.5 | 0.4 |  | 7 | 0.3 | 0.2 | 0.2 |  | 19 | 0.5 | 0.4 | 0.3 |  |
| **Social exclusion** | 38 | 6 | 0.3 | 0.2 | 0.2 |  | 4 | 0.3 | 0.2 | 0.2 |  | 6 | 0.3 | 0.2 | 0.2 |  |
| **Stigma experience** | 24 | 7 | 0.3 | 0.2 | 0.2 |  | 6 | 0.1 | 0.1 | 0.1 |  | 6 | 0.1 | 0.1 | 0.1 |  |
| **COMBINED** |  | **30** | **0.4** | **0.3** | **0.2** |  | **16** | **0.1** | **0.1** | **0.**1 |  | **28** | **0.3** | **0.2** | **0.2** |  |
| ***ATTAINABLE^c^*** |  | ***95*** | ***6.1*** | ***4.8*** | ***3.8*** |  | ***90*** | ***3.8*** | ***2.9*** | ***2.5*** |  | ***95*** | ***6.0*** | ***4.8*** | ***3.9*** |  |
| ***APC^d^*** |  | ***5%*** | ***0.3*** | ***0.3*** | ***0.2*** |  | ***5%*** | ***0.2*** | ***0.2*** | ***0.2*** |  | ***5%*** | ***0.3*** | ***0.3*** | ***0.2*** |  |

Note: ES- effectiveness of existing interventions at reducing the rate of risk factors. PAF – population attributable fraction; LYG –life years gained at specific ages from reducing a cause weighted by the ES; ^a^ Combined=comorbidity adjusted gain in life expectancy for specific determinants; ^b^ Lithium is prescribed for bipolar disorder, mainly. ^c^ Attainable= potential gain in life expectancy considering the combined effect of multiple risk factors; ^d^ APC – annual percentage change, together with amount of annual change (rounded data) in life expectancy.

**References**

1. Janney CA, Fagiolini A, Swartz HA, Jakicic JM, Holleman RG, Richardson CR. Are adults with bipolar disorder active? Objectively measured physical activity and sedentary behavior using accelerometry. J Affect Disord. 2014;152-154:498-504.

2. Janney CA, Ganguli R, Richardson CR, Holleman RG, Tang G, Cauley JA, et al. Sedentary behavior and psychiatric symptoms in overweight and obese adults with schizophrenia and schizoaffective disorders (WAIST Study). Schizophr Res. 2013;145(1-3):63-8.

3. Mangurian C, Newcomer JW, Modlin C, Schillinger D. Diabetes and Cardiovascular Care Among People with Severe Mental Illness: A Literature Review. J Gen Intern Med. 2016;31(9):1083-91.

4. Jackson JG, Diaz FJ, Lopez L, de Leon J. A combined analysis of worldwide studies demonstrates an association between bipolar disorder and tobacco smoking behaviors in adults. Bipolar Disord. 2015;17(6):575-97.

5. Vancampfort D, Stubbs B, Mitchell AJ, De Hert M, Wampers M, Ward PB, et al. Risk of metabolic syndrome and its components in people with schizophrenia and related psychotic disorders, bipolar disorder and major depressive disorder: a systematic review and meta-analysis. World Psychiatry. 2015;14(3):339-47.

6. Jakobsen AS, Speyer H, Norgaard HCB, Karlsen M, Hjorthoj C, Krogh J, et al. Dietary patterns and physical activity in people with schizophrenia and increased waist circumference. Schizophr Res. 2018.

7. Hunt GE, Malhi GS, Cleary M, Lai HM, Sitharthan T. Prevalence of comorbid bipolar and substance use disorders in clinical settings, 1990-2015: Systematic review and meta-analysis. J Affect Disord. 2016;206:331-49.

8. Viron MJ, Stern TA. The impact of serious mental illness on health and healthcare. Psychosomatics. 2010;51(6):458-65.

9. Green B, Young R, Kavanagh D. Cannabis use and misuse prevalence among people with psychosis. Br J Psychiatry. 2005;187:306-13.

10. O'Donoghue B, Roche E, Lane A. Neighbourhood level social deprivation and the risk of psychotic disorders: a systematic review. Soc Psychiatry Psychiatr Epidemiol. 2016;51(7):941-50.

11. Kwok W. Is there evidence that social class at birth increases risk of psychosis? A systematic review. Int J Soc Psychiatry. 2014;60(8):801-8.

12. Hawke LD, Parikh SV, Michalak EE. Stigma and bipolar disorder: a review of the literature. J Affect Disord. 2013;150(2):181-91.

13. Gerlinger G, Hauser M, De Hert M, Lacluyse K, Wampers M, Correll CU. Personal stigma in schizophrenia spectrum disorders: a systematic review of prevalence rates, correlates, impact and interventions. World Psychiatry. 2013;12(2):155-64.

14. Michalska da Rocha B, Rhodes S, Vasilopoulou E, Hutton P. Loneliness in Psychosis: A Meta-analytical Review. Schizophr Bull. 2018;44(1):114-25.

15. Gayer-Anderson C, Morgan C. Social networks, support and early psychosis: a systematic review. Epidemiol Psychiatr Sci. 2013;22(2):131-46.

16. O'Brien A, Fahmy R, Singh SP. Disengagement from mental health services. A literature review. Soc Psychiatry Psychiatr Epidemiol. 2009;44(7):558-68.

17. Mitchell AJ, Lawrence D. Revascularisation and mortality rates following acute coronary syndromes in people with severe mental illness: comparative meta-analysis. Br J Psychiatry. 2011;198(6):434-41.

18. Baller JB, McGinty EE, Azrin ST, Juliano-Bult D, Daumit GL. Screening for cardiovascular risk factors in adults with serious mental illness: a review of the evidence. BMC Psychiatry. 2015;15:55.

19. Patel MX, Bishara D, Jayakumar S, Zalewska K, Shiers D, Crawford MJ, et al. Quality of prescribing for schizophrenia: evidence from a national audit in England and Wales. Eur Neuropsychopharmacol. 2014;24(4):499-509.

20. Cipriani A, Hawton K, Stockton S, Geddes JR. Lithium in the prevention of suicide in mood disorders: updated systematic review and meta-analysis. BMJ. 2013;346:f3646.

21. Peckham E, Brabyn S, Cook L, Tew G, Gilbody S. Smoking cessation in severe mental ill health: what works? an updated systematic review and meta-analysis. BMC Psychiatry. 2017;17(1):252.

22. Ashdown-Franks G, Williams J, Vancampfort D, Firth J, Schuch F, Hubbard K, et al. Is it possible for people with severe mental illness to sit less and move more? A systematic review of interventions to increase physical activity or reduce sedentary behaviour. Schizophr Res. 2018.

23. Teasdale SB, Ward PB, Rosenbaum S, Watkins A, Curtis J, Kalucy M, et al. A nutrition intervention is effective in improving dietary components linked to cardiometabolic risk in youth with first-episode psychosis. Br J Nutr. 2016;115(11):1987-93.

24. Naslund JA, Whiteman KL, McHugo GJ, Aschbrenner KA, Marsch LA, Bartels SJ. Lifestyle interventions for weight loss among overweight and obese adults with serious mental illness: A systematic review and meta-analysis. Gen Hosp Psychiatry. 2017;47:83-102.

25. Hunt GE, Siegfried N, Morley K, Sitharthan T, Cleary M. Psychosocial interventions for people with both severe mental illness and substance misuse. Cochrane Database Syst Rev. 2013(10):CD001088.

26. Tidey JW. Using incentives to reduce substance use and other health risk behaviors among people with serious mental illness. Prev Med. 2012;55 Suppl:S54-60.

27. Tosh G, Clifton AV, Xia J, White MM. General physical health advice for people with serious mental illness. Cochrane Database Syst Rev. 2014(3):CD008567.

28. Vermeulen JM, van Rooijen G, van de Kerkhof MPJ, Sutterland AL, Correll CU, de Haan L. Clozapine and Long-Term Mortality Risk in Patients With Schizophrenia: A Systematic Review and Meta-analysis of Studies Lasting 1.1-12.5 Years. Schizophr Bull. 2018.

29. Lamontagne-Godwin F, Burgess C, Clement S, Gasston-Hales M, Greene C, Manyande A, et al. Interventions to increase access to or uptake of physical health screening in people with severe mental illness: a realist review. BMJ Open. 2018;8(2):e019412.

30. Liu NH, Daumit GL, Dua T, Aquila R, Charlson F, Cuijpers P, et al. Excess mortality in persons with severe mental disorders: a multilevel intervention framework and priorities for clinical practice, policy and research agendas. World Psychiatry. 2017;16(1):30-40.

31. Corrigan PW, Morris SB, Michaels PJ, Rafacz JD, Rusch N. Challenging the public stigma of mental illness: a meta-analysis of outcome studies. Psychiatr Serv. 2012;63(10):963-73.

32. Tsang HW, Ching SC, Tang KH, Lam HT, Law PY, Wan CN. Therapeutic intervention for internalized stigma of severe mental illness: A systematic review and meta-analysis. Schizophr Res. 2016;173(1-2):45-53.

33. McDonagh MS, Dana T, Selph S, Devine EB, Cantor A, Bougatsos C, et al. Treatments for Schizophrenia in Adults: A Systematic Review. AHRQ Comparative Effectiveness Reviews. Rockville (MD)2017.

34. Anderson K, Laxhman N, Priebe S. Can mental health interventions change social networks? A systematic review. BMC Psychiatry. 2015;15:297.

35. Beary M, Hodgson R, Wildgust HJ. A critical review of major mortality risk factors for all-cause mortality in first-episode schizophrenia: clinical and research implications. J Psychopharmacol. 2012;26(5 Suppl):52-61.

36. Callaghan RC, Veldhuizen S, Jeysingh T, Orlan C, Graham C, Kakouris G, et al. Patterns of tobacco-related mortality among individuals diagnosed with schizophrenia, bipolar disorder, or depression. J Psychiatr Res. 2014;48(1):102-10.

37. Bellavia A, Larsson SC, Bottai M, Wolk A, Orsini N. Fruit and vegetable consumption and all-cause mortality: a dose-response analysis. Am J Clin Nutr. 2013;98(2):454-9.

38. Chwastiak LA, Rosenheck RA, Desai R, Kazis LE. Association of psychiatric illness and all-cause mortality in the National Department of Veterans Affairs Health Care System. Psychosom Med. 2010;72(8):817-22.

39. Hjorthoj C, Ostergaard ML, Benros ME, Toftdahl NG, Erlangsen A, Andersen JT, et al. Association between alcohol and substance use disorders and all-cause and cause-specific mortality in schizophrenia, bipolar disorder, and unipolar depression: a nationwide, prospective, register-based study. Lancet Psychiatry. 2015;2(9):801-8.

40. Correll CU, Solmi M, Veronese N, Bortolato B, Rosson S, Santonastaso P, et al. Prevalence, incidence and mortality from cardiovascular disease in patients with pooled and specific severe mental illness: a large-scale meta-analysis of 3,211,768 patients and 113,383,368 controls. World Psychiatry. 2017;16(2):163-80.

41. Wimberley T, MacCabe JH, Laursen TM, Sorensen HJ, Astrup A, Horsdal HT, et al. Mortality and Self-Harm in Association With Clozapine in Treatment-Resistant Schizophrenia. Am J Psychiatry. 2017;174(10):990-8.

42. Lewitzka U, Severus E, Bauer R, Ritter P, Muller-Oerlinghausen B, Bauer M. The suicide prevention effect of lithium: more than 20 years of evidence-a narrative review. Int J Bipolar Disord. 2015;3(1):32.

43. Copeland LA, Zeber JE, Wang CP, Parchman ML, Lawrence VA, Valenstein M, et al. Patterns of primary care and mortality among patients with schizophrenia or diabetes: a cluster analysis approach to the retrospective study of healthcare utilization. BMC Health Serv Res. 2009;9:127.

44. Mitchell AJ, Malone D, Doebbeling CC. Quality of medical care for people with and without comorbid mental illness and substance misuse: systematic review of comparative studies. Br J Psychiatry. 2009;194(6):491-9.

45. Bergamo C, Sigel K, Mhango G, Kale M, Wisnivesky JP. Inequalities in lung cancer care of elderly patients with schizophrenia: an observational cohort study. Psychosom Med. 2014;76(3):215-20.

46. Kilbourne AM, Morden NE, Austin K, Ilgen M, McCarthy JF, Dalack G, et al. Excess heart-disease-related mortality in a national study of patients with mental disorders: identifying modifiable risk factors. Gen Hosp Psychiatry. 2009;31(6):555-63.

47. Barnes LL, de Leon CF, Lewis TT, Bienias JL, Wilson RS, Evans DA. Perceived discrimination and mortality in a population-based study of older adults. Am J Public Health. 2008;98(7):1241-7.
